# Creating a win-win for the health system and health Profession’s education: a direct observation clinical experience with feedback iN real-time (DOCENT) for low acuity patients in the emergency department

**DOI:** 10.1186/s12909-022-03133-z

**Published:** 2022-01-27

**Authors:** Alison S. Clay, Erin R. Leiman, Brent Jason Theiling, Yao Song, Blanca Blanca Iris Padilla, Nicholas M. Hudak, Ann Michelle Hartman, Jeffrey M. Hoder, Kathleen A. Waite, Hui-Jie Lee, Edward G. Buckley

**Affiliations:** 1 Department of Medicine, School of Medicine, Duke University School of Medicine, 8 Searle Center Drive, TSCHE 1074, Durham, NC 27710 USA; 2 Department of Surgery, School of Medicine, Duke University, Durham, USA; 3 Department of Biostatistics and Bioinformatics, School of Medicine, Duke University, Durham, USA; 4 School of Nursing, Duke University, Durham, USA; 5 Department of Neurology, School of Medicine, Duke University, Durham, USA; 6 Department of Orthopedic Surgery, School of Medicine, Duke University, Durham, USA; 7 Department of Ophthalmology, School of Medicine, Duke University, Durham, USA

**Keywords:** Undergraduate medical education, Health professions education, Direct observation, Feedback, Entrustable professional activities, Interprofessional collaboration, Learning climate

## Abstract

**Background:**

Clinical education across the professions is challenged by a lack of recognition for faculty and pressure for patient throughput and revenue generation. These pressures may reduce *direct* observation of patient care provided by students, a requirement for both billing student-involved services and assessing competence. These same pressures may also limit opportunities for interprofessional education and collaboration.

**Methods:**

An interprofessional group of faculty collaborated in a sequential quality improvement project to identify the best patients and physical location for a student teaching clinic. Patient chief complaint, use of resources, length of stay, estimated severity of illness and student participation and evaluation of the clinic was tracked.

**Results:**

Clinic Optimization and Patient Care: Five hundred and thirty-two emergency department (ED) patients were seen in the first 19 months of the clinic. A clinic located near the ED allowed for patients with higher emergency severity index and greater utilization of imaging**.** Patients had similar or lower lengths of stay and higher satisfaction than patients who remained in the ED (*p* < 0.0001). In the second clinic location, from October 2016–June 2019, 644 patients were seen with a total of 667 concerns; the most common concern was musculoskeletal (50.1%).

Student Interprofessional Experience: A total of 991 students participated in the clinic: 68.3% (*n* = 677) medical students, 10.1% (*n* = 100) physician assistant students, 9.7% (*n* = 96) undergraduate nursing students, 9.1% (*n* = 90) physical therapy students, and 2.8% (*n* = 28) nurse practitioner students. The majority (74.5%, *n* = 738) of student participants worked with students from other professions. More than 90% of students reported that faculty set a positive learning environment respectful of students. However, 20% of students reported that faculty could improve provision of constructive feedback.

Direct Observation: Direct observation of core entrustable professional activities for medical students was possible. Senior medical students were more likely to be observed generating a differential diagnosis or management plan than first year medical students.

**Conclusions:**

Creation of a DOCENT clinic in the emergency department provided opportunities for interprofessional education and observation of student clinical skills, enriching student experience without compromising patient care.

## Introduction

Clinical education demands direct observation of student skills, including interprofessional collaboration and teamwork, which is required by all the major accrediting agencies for health professions education [1–4]. In May 2014, the American Association of Medical Colleges identified an additional 12 core entrustable professional activities for entering residency that require direct observation for skill assessment and evaluation [5]. Additionally, direct observation is a critical component of coaching as it provides small steps of change to improve care in the next encounter [6, 7]. Despite these requirements, direct observation of skills and interprofessional collaboration remain difficult.

Barriers to direct observation of student clinical skills include both systemic and interpersonal barriers. Faculty and trainees often cite patient volume, lack of dedicated time and inefficient care by trainees as precluding good educational experiences [6–12]. Conflicts often arise, particularly in private practices, regarding who to prioritize - patients or students - which can lead to loss of income [8, 11, 13]. Failure of professional programs to provide clear expectations, define educational experiences and offer standardized assessments contributes to poor faculty preparation [8, 9, 11, 13, 14]. Students describe experiences that benefit the patient and not the student (i.e. “work without education”) or that result in “shadowing” only [8, 9, 11, 13, 14]. An absence of physical space and patient discomfort as well as poor faculty development and insufficient financial and professional recognition further hampers faculty participation in education [8–11, 13, 15]. Lack of clarity about who drives direct observation and feedback and/or lack of standardized assessments contributes to both learner and faculty disappointment [11, 13]. When given, feedback does not necessarily contain certain critical components, such as being timely, specific, based upon observation, appropriate to student level, providing what is needed to improve, and motivating [16, 17]. Perhaps because of this negative learning environment, learners report increasing rates of neglect and mistreatment [14, 18, 19].

While interprofessional education struggles with similar problems, some of the issues experienced are unique. Different training durations, varying schedules for curricula, and use of different clinical training sites make it difficult for learners from varying health professions programs to practice together for single sessions [20, 21]. When these sessions do occur, students do not work together again as part of a class or clinic experience [20]. Different professional programs prioritize sessions differently and may not agree if sessions should be “within” the curricula (or required) or an extracurricular activity. Finally, faculty may not receive adequate training, have limited knowledge of other professions [12, 20–22] or are not provided with standardized assessments critical for provision of timely and specific feedback [20–22]. Faculty and students may express bias towards other professions that further hampers successful collaborations [12, 20]. Finally, lack of administrative support and financing contribute to lack of interprofessional education [12, 20, 21].

One proposed solution for interprofessional education and collaboration is student-run free clinics. By relying on interested students and faculty, these clinics avoid bias towards interprofessional education [23] and offer peer-to-peer teaching, which is considered an asset [22]. Students care for complex patients and better understand the role of teams in patient care [24]. As extracurricular activities, the clinics allow the freedom for each professional school to opt-in, allowing for wider participation of professions [25–29] but no academic credit is provided for student involvement [25]. However, clinics are infrequent, often operating once a month or once a week, offer care outside of a health system, and struggle with faculty staffing, funding and sustainability as well as liability coverage [25, 30]. Clinics are critiqued for potentially taking advantage of vulnerable populations or for offering care provided by trainees to patients who cannot get access to care any other way [31, 32]. Student-run free clinics can reduce emergency department (ED) utilization by patients who would choose this avenue for treatment without access to the clinics [33].

The use of the emergency department for either interprofessional students or medical students is not often described in the literature, though this is a location with a near constant supply of patients and care is both interprofessional and multi-disciplinary. Patients have a variety of concerns and are often undifferentiated, allowing for rich discussions of differential diagnosis. Inclusion of students in emergency department clerkships have shown that patients have longer lengths of stay and increased use of resources [34].

Our goals for this project were to 1) create a regularly scheduled student educational experience within the health system emergency department that provided patient care as good as or better than similar care provided in our emergency department and 2) create a clinic with dedicated and trained faculty that provided interprofessional education as often as possible (given the different needs of the health professions programs), and 3) provide students with direct observation of clinical skills without the time pressures usually associated with clinical education.

The purpose of this paper is to describe how we developed an interprofessional student clinic in an emergency department of large academic health system that allowed (at best) interprofessional collaborative student experiences and (at least) opportunity for direct observation of medical students’ clkinical skills. We demonstrate how this clinic served as a “win” for the health system and patients and a “win” for the health professions programs so that others might build similar experiences for their students.

## Methods

### Clinic creation and optimization

A Direct Observation Clinical Experience with feedback iN real-Time (DOCENT) was created with leadership from the doctor of medicine (MD), physician assistant (PA), and the School of Nursing, including the nurse practitioner (NP) programs and accelerated bachelor of science in nursing (ABSN). The group sought to provide care to patients already seeking medical care within our health system that would benefit from a teaching team and thus, it included emergency department patients with a low emergency severity index (ESI) [35, 36]. This scores signifies patients with low acuity of illness not expected to consume significant healthcare resources. These patients often experienced long wait times and sometimes left without being seen; caring for this patent population would benefit the patients as well as the health system.

The physical location for DOCENT (December 2015–December 2016) was an underutilized outpatient clinic space already staffed with nurses (an infusion clinic that operated until 9:00 pm nightly). Therefore, from 5 pm–9 pm, patients for DOCENT were triaged in the emergency department, assigned an emergency severity index, and a medical screening exam was performed by emergency medicine physicians. Inclusion and exclusion criteria for patients included: non-pregnant patients > age 18, an emergency severity index score > 3, and not expected to require imaging. Eligible patients were consented to participate in DOCENT or could choose to remain in the emergency department. Once consented, patients were walked to the clinic space by DOCENT students and faculty. Clinic faculty logged all patients’ chief concerns. Patient presence in the clinic was tracked via the electronic health record when a patient was moved to a bed affiliated with DOCENT.

In January 2017, the clinic was moved to a physical location closer to the emergency department in a space that was only utilized during daytime hours and thus open for evening use. The closer proximity to the emergency department expanded the number of eligible patients by making it easier to see patients with higher emergency severity index scores and those who might need radiologic imaging. Nurses from the emergency department provided nursing coverage. During this phase of clinic optimization, the nights of operation changed from Mondays to Thursdays. Many patients arriving at the emergency department on Friday night were sent to the emergency department by outlying clinics for admission, consultation, or additional testing. Consequently, completing encounters during DOCENT hours was difficult. Further, this freed up funds that could be used to support a health system credentialled nurse who could work in the clinic and supervise undergraduate level nursing students.

Faculty facilitating DOCENT selected the patients, completed medical screening exams, and consented the patients. Patients were selected from the emergency department electronic triage board based on 1) their eligibility (as described above), 2) faculty belief that the patient’s encounter could be completed before the end of the DOCENT clinic hours, 3) students expressed learning objectives, and 4) faculty comfort with the patient’s stated concern. Once selected, faculty approached the patient and consented the patient to participate in the clinic. If they declined, patients would remain in the standard triage queue for the emergency department. Once selected and consented, patients were escorted to the clinic.

DOCENT patients were tracked manually via a clinic log. After the 300th patient, the types of concerns were reviewed and categorized retrospectively by leadership. Thereafter, the category of patient concern was logged prospectively. Patient-specific data were tracked by the health system for quality improvement purposes and subsequently reviewed for this study, including emergency severity index, length of stay, use of diagnostic imaging, and payer. Clinic patients were identified after being “moved” electronically in the electronic health record to the DOCENT “space”. ED patients were selected as a comparison group if they had a similar severity of illness and presented during weekdays between 3:00 pm and 7:00 pm, the equivalent period of time during which DOCENT patients were triaged. Patient care experiences in DOCENT vs. the emergency department ED were evaluated using a Kruskal-Wallis test to compare length of stay and Chi-square tests to compare distributions for illness severity and number of patients receiving radiologic imaging.

During the first year, patients seen as part of DOCENT were contacted by telephone to assess satisfaction with the care provided. Subsets of the clinic population and comparison patients from the emergency department were asked to complete standard surveys sent by the health system after care was completed. Four Likert-type questions were asked with possible responses of ‘very poor’, ‘poor’, ‘fair’, ‘good’, and ‘very good’: 1) How well was your team’s concern for your comfort? 2) How well did the team take time to listen to you? 3) How well were you kept informed of delays? and 4) How likely would you be to recommend this experience? Patient satisfaction between DOCENT and the emergency department were evaluated using Fisher’s exact tests.

### Faculty support and responsibilities for DOCENT

The School of Medicine paid physician faculty to staff DOCENT at a rate commensurate to half of a clinic day. All faculty chose to work in addition to their pre-existing clinical responsibilities. During academic year 2017–2018, 13 faculty worked in the clinic, including five emergency medicine trained faculty, one family medicine and community health provider, and seven faculty trained in internal medicine or an internal medicine subspecialty. The number of shifts were equally divided between emergency medicine and non-emergency medicine faculty. The School of Medicine also paid an emergency department nurse (commensurate to their emergency department hourly rate plus overtime) to staff the clinic and provide interprofessional perspectives on care. Like faculty, nurses worked in the clinic in addition to their scheduled emergency department shifts. The physician assistant program encouraged their emergency medicine faculty to precept in the clinic 1 day a week and provided funds to pay for these extra shifts. The nurse practitioner and physical therapy programs each identified one faculty to work in the clinic with students and recognized this work as part of that individual’s academic responsibility. Two faculty physicians served as “Co-course Directors” for DOCENT and received an academic time adjustment and partial time from a coordinator. The course directors and coordinator scheduled students and faculty, developed orientation materials, and supported faculty development. Revenue generated from patient encounters was received by the health system and used to support clinic infrastructure/emergency department function.

All faculty completed onboarding and ongoing faculty development. Faculty development consisted of reviewing the student modules (as described above), shadowing a faculty member working in clinic, and twice-yearly faculty development sessions. During the yearly faculty development sessions, faculty were provided with their evaluations as well as students’ overall evaluation of clinic. There was also structured time for small group discussion on teaching strategies. Additionally, there were teaching sessions on a topic of their choosing (for example, suturing and basic bedside ultrasound).

Faculty provided verbal and written feedback for each session, during clinic debriefing and in a short online survey using Qualtrics™ respectively. Faculty were asked to answer two free-response questions: 1) What is one piece of reinforcing feedback you can provide and 2) what is one piece of constructive feedback you can provide. Once completed, the survey was automatically emailed to the student. Student’s completed their evaluation of the clinic experience asynchronous to receipt of this formal provision of feedback.

#### Student participation and evaluation

After confirming clinic location, operations and faculty, student participation was optimized. For Academic Year 2017–2018 (October 2017–June 2018), doctor of medicine students in Year 1 (preclinical year) and Year 3 (research year) were required to attend once during the academic year. Doctor of medicine students in Year 4 (clinical year) could attend as part of an elective. Physician assistant students in Year 2 (clinical year) participated as part of their emergency medicine rotation. Doctor of physical therapy students, pre-licensure nursing students and nurse practitioner students participated voluntarily at different times throughout their training. For the purpose of appropriate supervision, doctor of physical therapy students could only participate when a faculty member was also present (as a volunteer) in the clinic (i.e., medical doctors cannot supervise physical therapists). Similarly, undergraduate nursing students could only work in the clinic on nights when an emergency department nurse was present in the clinic to supervise their scope of practice. Physician faculty supervised all care provided, and supervised doctor of medicine, physician assistant and nurse practitioner students in the clinic on every night the clinic was in operation.

All students participating in the clinic were required to complete two 15-min orientation modules. One video compared and contrasted health professions training (including length of training, clinical requirements, need for post-graduate training). The second video introduced the students to the purpose of the clinic (interprofessional education and provision of direct observation and real-time feedback). Types of feedback (formative, summative, reinforcing and constructive feedback) were discussed in the orientation modules. Students attested to completing the modules. During a pre-clinic huddle, students learned about the patient consent process, clinical logistics, and selection of eligible patients. Most importantly, during this time, all students were asked to share one goal for their personal learning and this was when there would be a discussion regarding division of patient care responsibilities and the importance of inclusion of the patient as teacher. A teaching triangle structure was utilized through the inclusion of the patient as a teacher; as all learner teaching and patient care and team discussions occurred in the same room as the patient [37]. At the conclusion of the patient encounter, the team would debrief the experience and this included students providing feedback to faculty and faculty providing feedback to students. Students and faculty were expected to also complete a formal evaluation of their clinic experience through emailed surveys after clinic.

Student participation was tracked formally through these written evaluations. All students received instructions and reminders to complete evaluations but evaluations were mandatory for the medical students participating as a requirement of their formal curriculum. A coordinator tracked completion of the medical students’ shifts and evaluations. All students were asked if they worked with students or faculty from another profession. Students were also asked to rate whether faculty created a positive learning climate respectful of the learner and provided reinforcing or constructive feedback using a Likert scale (no, somewhat, yes). Additionally, clinical students were asked if they had the opportunity to supervise preclinical students. The clinic emphasized feedback and learning climate since these are questions medical students are asked on surveys of their clerkships and at the end of medical school as part of ongoing accreditation by the American Association of Medical Colleges.

Given the emergence of the American Association of Medical College’s thirteen entrustable professional activities [5], medical students were also asked if they were directly observed obtaining a history, performing a physical exam, and creating differential diagnosis and/or management plans (yes/no). Since multiple students worked in the clinic together, it was possible for one student to report not being directly observed for one aspect of the encounter because another student was responsible for that part of the encounter. To compare medical students’ experiences in DOCENT, survey responses for medical students were divided into preclinical (1st year) and clinical (3rd year or 4th year) students.

A two-sided significance level of 0.05 was used for all statistical tests between health professions or medical school groups without a multiple comparison adjustment. Statistical analyses were performed using SAS version 9.4 (SAS Institute, Inc., Cary, NC, USA) and R version 3.4.0 (R Core Team, Vienna, Austria) for Windows. This study was reviewed and received exempt status from the Duke Health Institutional Review Board.

## Results

### Patient care

In the first 18 months of the clinic, a total of 532 patients were seen as part of the direct observation experience for medical students and a total of 11,127 emergency department patients were selected as a comparison group. The DOCENT patients had a shorter or analogous length of stay when compared to emergency department patients (mean 4.5 vs. 6.4 h, *p* < 0.0001) with similar acuity and time of presentation to the emergency department.

**Fig. 1:**
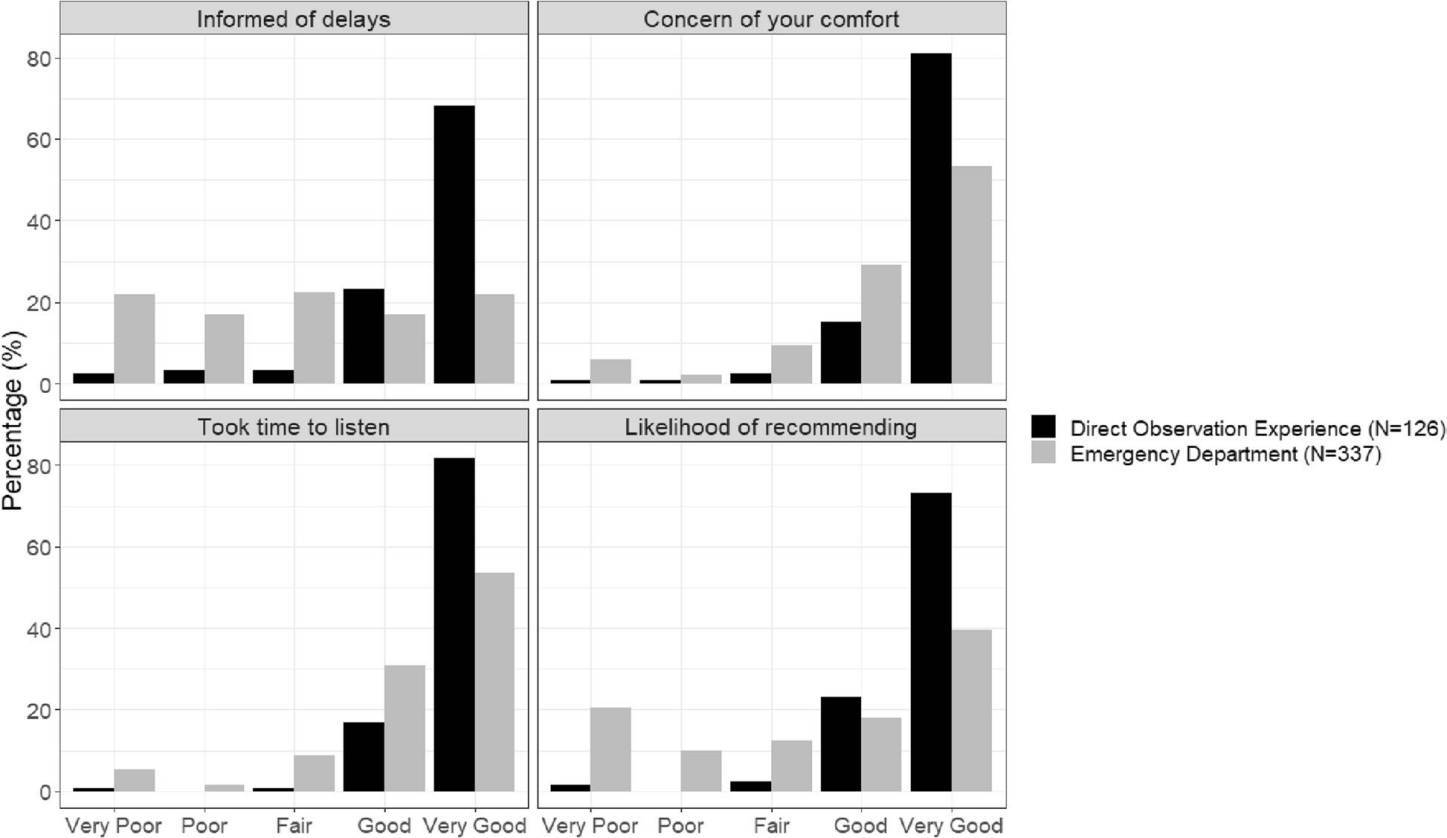
Satisfaction of DOCENT patients compared to patients who remained in the emergency department

DOCENT patients reported higher satisfaction than those patients who remained in the ED. Comparisons were initially made with completed paper surveys of 14 DOCENT patients with 336 emergency department patients who completed the same survey. The number of patient satisfaction surveys from DOCENT was small because DOCENT patients represent a very small percentage of all patients seen in the emergency department. Therefore, we also compared DOCENT patient responses from a telephone survey with the responses from emergency department patients. Comparisons were made between 337 emergency department patients who completed paper patient satisfaction surveys and 126 DOCENT clinic patients who completed follow-up phone calls with the same questions (see Fig. 1). This was the same trend seen when comparing 14 DOCENT patients with the 336 emergency department patients, however, this N was too small to perform statistical analysis.

**Fig. 2:**
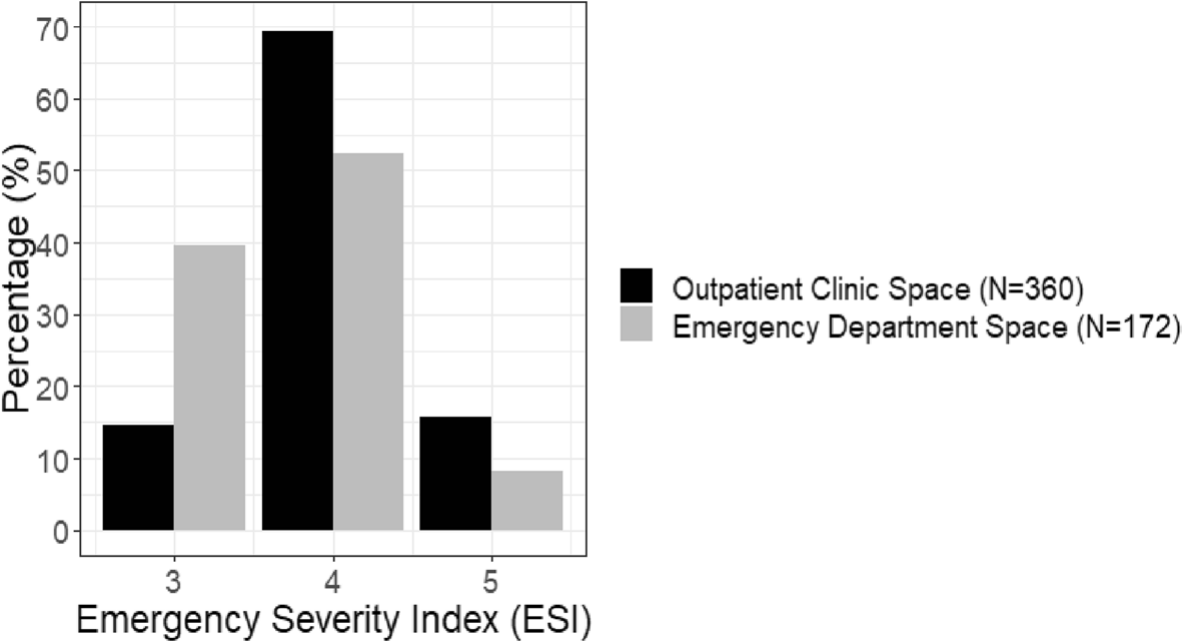
Emergency severity index for DOCENT patients seen at two different physical locations

When DOCENT was moved from a traditional outpatient-type clinic space to a location closer to the emergency department, higher acuity patients were seen (see Fig. 2). Although acuity increased, patient length of stay was similar between the clinic spaces further away from and closer to the emergency department (M = 4.6 h, SD = 2.6 h) vs. (M = 4.5 h, SD = 2.5 h), *p* = 0.9306). A greater percentage of patients seen in the second location (closer to the emergency department) required radiologic investigations (37.6% (64/172) vs. 17.4% (60/360), *p* < 0.0001). The payor distribution for patients of various emergency severity index level and location (emergency department (*n* = 20,847) versus DOCENT (*n* = 536) clinic) demonstrates that patients seen in the clinic have a diverse set of payors, including self-pay, Medicare, Medicaid and private insurance (Table 1).

**Table 1:** Payer information for patients seen in the emergency department and DOCENT clinic

Acuity	Emergency Department				DOCENT Clinic			
	2	3	4	5	2	3	4	5
	*N* = 9720	*N* = 7900	*N* = 2834	*N* = 393	*N* = 4	*N* = 121	*N* = 340	*N* = 71
**Payer Information**								
Self-Pay	920 (9.5%)	1484 (18.8%)	940 (33.2%)	162 (41.2%)	0 (0.0%)	31 (25.6%)	128 (37.6%)	30 (42.3%)
Medicaid	1165 (12.0%)	1259 (15.9%)	516 (18.2%)	83 (21.1%)	0 (0.0%)	22 (18.2%)	68 (20.0%)	9 (12.7%)
Medicare	3483 (35.8%)	1788 (22.6%)	314 (11.1%)	34 (8.7%)	4 (100.0%)	11 (9.1%)	32 (9.4%)	5 (7.0%)
Private Insurance Or Others	4152 (42.7%)	3369 (42.6%)	1064 (37.5%)	114 (29.0%)	0 (0.0%)	57 (47.1%)	112 (32.9%)	27 (38.0%)

After the final location was established (October 2016 to June 2019, 644 patients were evaluated in the direct observation clinical experience with 667 chief complaints. The most common concerns were musculoskeletal (MSK) (see Table 2). The number of musculoskeletal complaints evaluated by health care teams may be influenced by participation of students and faculty from the doctor of physical therapy program.

**Table 2:** Types of patient concerns seen from October 2016 to June 2019 (*n* = 667)

Category	Number of Patients	% of Overall Concerns
**Musculoskeletal**	339	50.1%
**Head, eyes, ears, nose or throat (HEENT)**	89	13.3%
**Gastrointestinal (GI)**	67	10%
**Lower Respiratory**	55	8.3%
**Dermatologic**	43	6.5%
**Genitourinary (GU)**	32	4.8%
**Chronic Conditions**	20	3.0%
**Other**	22	3.3%

### Student experience

From October 2016 to June 2019, a total of 991 students participated in the clinic: 68.3% (*n* = 677) medical students, 10.1% (*n* = 100) physician assistant students, 9.7% (*n* = 96) prelicensure nursing students, 9.1% (*n* = 90) doctor of physical therapy students, and 2.8% (*n* = 28) nurse practitioner students. 74.5% (*n* = 738) of students had an interprofessional student experience working with students from another profession while 24.5% (*n* = 252) of students experienced clinic with all participants from the same profession or only *faculty* from another profession. In these clinics, 70% (*n* = 172) were single profession (students and faculty) and the remaining 30% (*n* = 80) worked with similar students but had a faculty member from a different profession. The majority of students affected by this were medical students, (97.2%, *n* = 245).

**Fig. 3:**
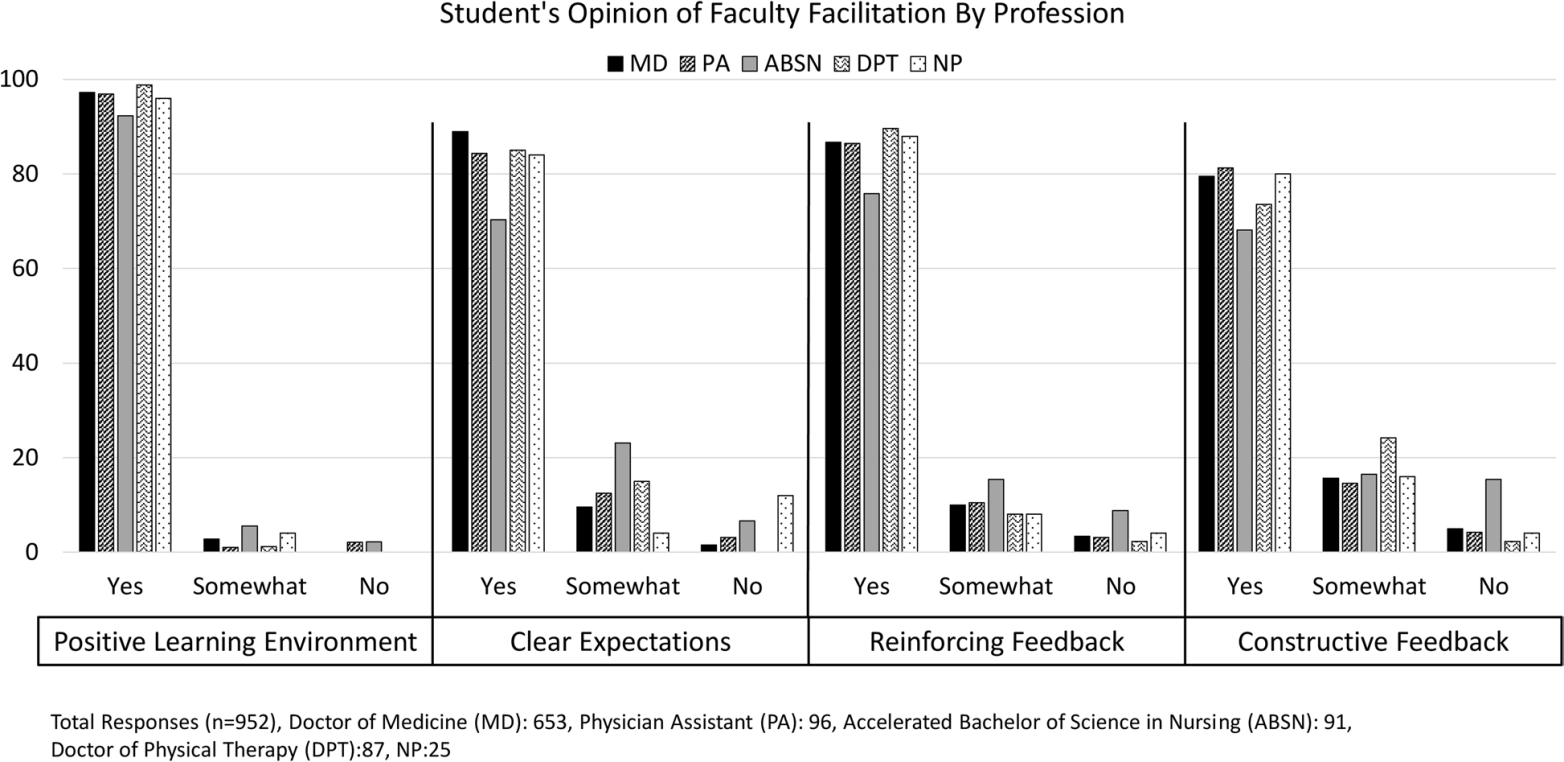
Student opinions (October 2016–June 2019) on key aspects of faculty facilitation (percentage of students responding)

From October 2016 to June 2019, 952 students provided feedback on faculty facilitation of the experience (see Fig. 3). Overall, students felt that faculty created a positive learning climate respectful of the learner. A greater percentage of undergraduate nursing students reported that faculty provided clear expectations “somewhat”. The greatest area for improvement for all professions was providing constructive feedback to learners.

**Fig. 4:**
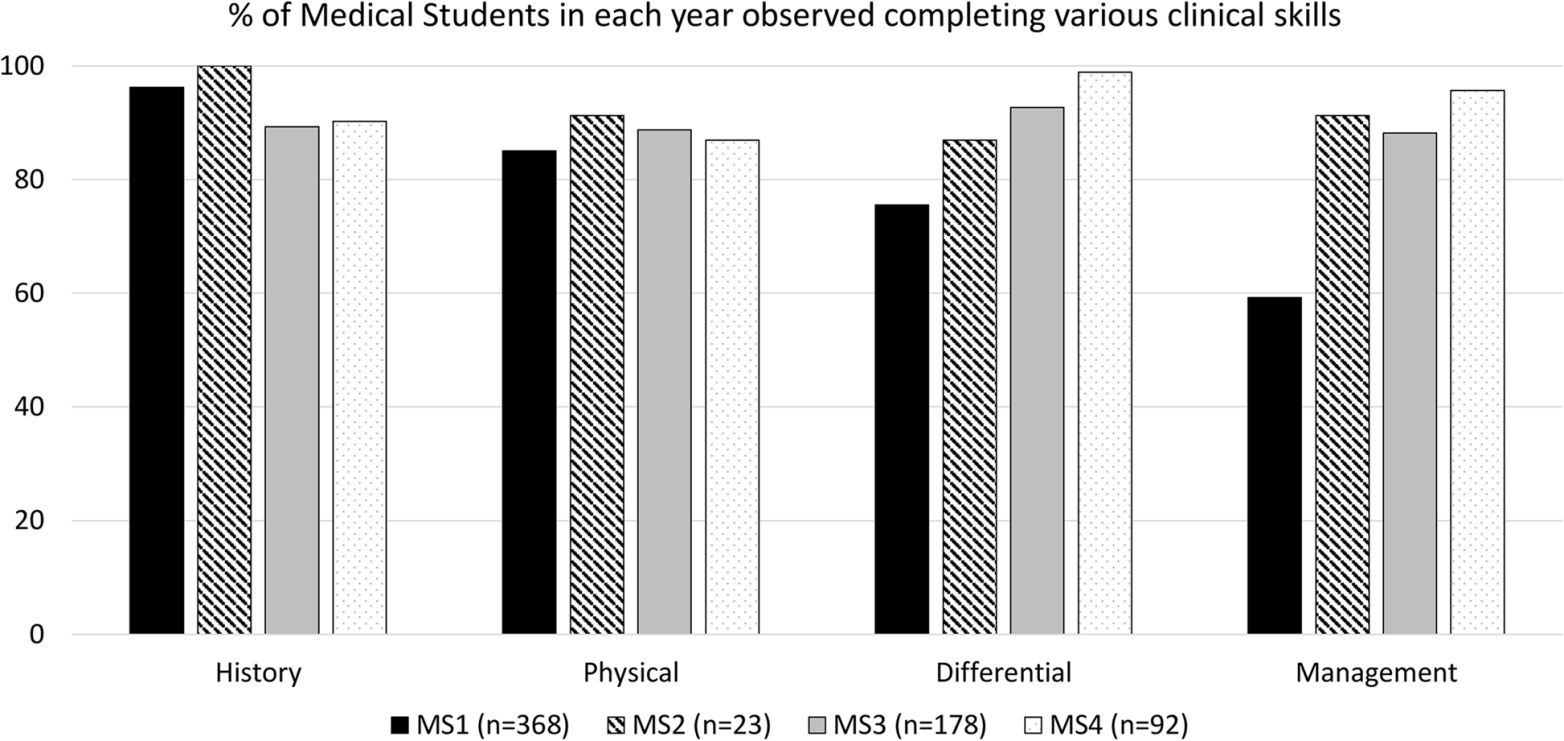
Percentage of medical students in each year observed completing various clinical skills

Medical students (1st and 3rd year) were required to attend the DOCENT clinic and worked closely with faculty from the School of Medicine who were explicitly advised to directly observe completing a history, physical exam, generating a differential diagnosis, or composing a management plan; these are entrustable professional activities for entering residency. The percentage of students from each class observed completing each skill is noted in Fig. 4. Since students were often paired with another student, they divided activities and a single student might not be observed completing part of the patient care encounter. First-year medical students were less likely than other students to participate in differential diagnosis or creation of management plans. The vast majority (94%) of clinical medical students (2nd, 3rd and 4th year) had the opportunity to participate in peer-to-peer teaching.

## Discussion

We have successfully created a DOCENT teaching clinic that benefits patients, students and health professional programs without adversely impacting the health system. The clinic also avoids many of the downfalls associated with other interprofessional experiences, such as student-run free clinics.

The DOCENT clinic represents a win for patients. Patients seen in the clinic have similar or lower wait times than other patients in the emergency department. Patients seen in DOCENT consent to participation and if they decline care, still receive the standard of care for emergency department patients. Patients from all payor groups are eligible for the clinic and out data suggests that patients with all payors take advantage of the clinic.

The DOCENT clinic is also a win for students and several of Duke’s health professions programs. Most students are able to experience an authentic team-based patient care experience in a positive learning environment that is respectful of students. Professional programs can provide interprofessional experiences for their students and have students directly observed completing key clinical skills. While a single observed encounter cannot adequately judge entrustability for students, these observations could contribute to a student portfolio that allows a clinical competency committee to evaluate students over time. Professional programs provide financial support for the teaching faculty, however, infrastructure for the clinic, including availability of radiology, laboratory services, pharmacists, interpreters, and social workers, is provided by the health system. This does not “cost” the health system as these patients would otherwise still be using these services in the emergence department.

The clinic’s success depended on four key features. First, it is regularly and predictably scheduled by a part-time administrator. This required the commitment of one professional program (the doctor of medicine program) to regularly schedule students (whose participation was mandatory), pay for recruited faculty, and ensure enough faculty to staff the clinic consistently. The inclusion of a part-time administrator allowed the oversight of student scheduling so that no clinic night had too many or too few students.

Second, the clinic is flexible. It allows professional programs with different durations of training, different numbers of students, and different geographic locations to participate on their own terms. The doctor of medicine’s flexibility to see the clinic as dual-purposed is also important. When possible, this allowed for interprofessional experiences, but when students were not practicing with a group from another profession, the school saw value in the opportunity to directly observe students in the setting of the new core entrustable professional activities for entry to residency. While a single decision of entrustment is not particularly useful (because skill may change as patient complexity changes, or skill may not be consistent), the decreasing continuity of supervisors and evaluation based on indirect data places importance to the data we could collect on students in the DOCENT clinic. This data could be used with other data for clinical competency committees.

The third component of success for the DOCENT clinic was having a location adjacent to the emergency department. This allowed for use of the emergency department’s infrastructure, including availability of respiratory therapists, pharmacists and notably, radiology. The access to radiology allowed faculty to see a greater number of patients with acute musculoskeletal needs. Inclusions of these patients made it easier to include doctor of physical therapy students and faculty.

Finally, the dedicated and supported faculty was essential. These faculty committed not only to teaching in the clinic, but to participating in faculty orientation, shadowing a provider in clinic and participating (as part of their reimbursement) in bi-annual faculty development sessions. Faculty found participation beneficial not only financially but also because the authentic teaching experiences were not pressured by time or patient throughput. Faculty truly valued the opportunity to learn from one another during faculty development sessions and most importantly, in clinic sessions staffed by more than one provider.

These early wins provide opportunity for improvement and future direction as well. First, the clinic can expand the professions currently working in clinic. For example, pharmacy students and social work students could be added and more time could be spent on management plans, social support, etc. Clinic faculty could be better trained to improve their ability to provide constructive feedback in person to students, especially students of different professions. Additionally, providers could be provided with more direction on how to give group feedback as well as individual feedback in front of their peers. We could also more formally review (qualitatively and by individual feedback to faculty) the reinforcing and constructive feedback provided to students to review themes and to improve individual faculty skill in providing feedback. Further work could also characterize the quality and types of feedback provided to students to better understand the similarities and differences in feedback students receive in this model of clinical education compared to their more traditional clinical teaching environments.

Faculty could improve supervision of the undergraduate nursing students. These students had lower scores on being provided clear expectations and provision of feedback than other professional students. Currently, those students are supervised by nurses from within the health system, not faculty from their professional school. Additional training for the health system nurses, greater involvement of clinic faculty from other professions in guiding the emergency department nurse, or faculty contribution from the School of Nursing who are also credentialed to work in the emergency department might improve these scores.

Finally, the clinic could work to increase opportunities for teams to work together again, later in the year. This would allow faculty to see growth of students over time (making entrustment decisions easier) and potentially strengthen the interprofessional experience for students. Student teams could also be scheduled to work together in other locations- on an inpatient rotation, at a nursing home, or an outpatient clinic. Similarly, a clinic elective could be approved by all the health professions programs allowing students to participate more in clinic leadership and/or in more frequent participation in the clinic; a multi-professional student leadership team (created as part of an elective offered to all students) could increase student participation and student satisfaction in the clinic.

### Study limitations

There are limitations to this study. Length of stay data were likely impacted by selection bias as faculty selected patients within their scope of practice and whose encounters were estimated to be completed during a clinic session. This may have resulted in seeing patients with less complicated concerns than other patients who had the same emergency severity index, thus affecting length of stay. Individual faculty may have influenced clinic data as well. For example, patient selection may vary based on the background of the faculty and thus, their experience and comfort with the various concerns that could be encountered in the emergency department waiting room. Each faculty provider has his or her own tolerance for risk. For example, one provider may order more radiologic or laboratory tests for a given complaint than others would for the same issue. Patient satisfaction data were impacted by response rates, including only those patients who took phone calls or completed written satisfaction surveys. Additionally, the difference in survey modality (phone call vs. written) could skew the response data. Student evaluations of the clinic may have been by impacted by whether their participation (and completion of evaluations) was mandatory (as was the case for first- and third-year medical students), or optional as was the case for the remaining students. Students’ evaluation of clinic may not have incorporated the formal feedback provided to them individually by faculty through their written personal evaluation. Faculty may have been less likely to provide reinforcing feedback in front of other students but still included this feedback in their written evaluation; feedback the student may not have received prior to completing their evaluation.

## Conclusions

By aligning the needs of the low acuity patients from the emergency department with the availability of interprofessional students and teaching faculty, and utilizing already provided for emergence department resources, we have successfully created a regularly scheduled and sustainable teaching clinic for students.

## Data Availability

The datasets used and/or analyzed during the current study are available from the corresponding author upon reasonable request. Quality improvement health system level data (about emergency severity index, length of stay) that contain potentially identifiable patient information is not able to be shared.

## Glossary

AAMC: American Association of Medical Colleges
CMS: Center for Medicare and Medicaid Services
DOCENT: Direct Observation Clinical Experience with feedback iN real Time
ED: Emergency Department
EHR: electronic health record
EM: Emergency Medicine
ESI: estimated severity of illness
EPA: entrustable professional activity
LOS: length of stay
PA: Physician Assistant
MD: Doctor of Medicine
MSK: musculoskeletal
NP: Nurse Practitioner
RVU: relative value unit
